# Emergence of carriage of CTX-M-15 in faecal *Escherichia coli* in horses at an equine hospital in the UK; increasing prevalence over a decade (2008–2017)

**DOI:** 10.1186/s12917-019-2011-9

**Published:** 2019-07-29

**Authors:** C. M. Isgren, T. Edwards, G. L. Pinchbeck, E. Winward, E. R. Adams, P. Norton, D. Timofte, T. W. Maddox, P. D. Clegg, N. J. Williams

**Affiliations:** 1 Institute of Infection and Global Health, University of Liverpool, Neston, England; 2 Research Centre for Drugs and Diagnostics, Liverpool School of Tropical Medicine, Liverpool, England; 3 Institute of Veterinary Science, University of Liverpool, Neston, England; 4 Department of Musculoskeletal Biology, Institute of Ageing and Chronic Disease, University of Liverpool, Liverpool, England

**Keywords:** ESBL-producing *E. coli*, Melt curve analysis, Multidrug resistance, High resolution melt, Real time PCR, CTX-M-1 group

## Abstract

**Background:**

This study investigated changes over time in the epidemiology of extended-spectrum β-lactamase (ESBL) producing *Escherichia coli* within a single equine referral hospital in the UK. Faecal samples were collected from hospitalised horses in 2008 and 2017, processed using selective media and standard susceptibility laboratory methods. A novel real-time PCR with high resolution melt analysis was used to distinguish *bla*_CTX-M-1_ and *bla*_CTX-M-15_ within CTX-M-1 group.

**Results:**

In 2008, 457 faecal samples from 103 horses were collected, with ESBL-producing *E. coli* identified in 131 samples (28.7, 95% CI 24.6–33.1). In 2017, 314 faecal samples were collected from 74 horses with ESBL-producing *E. coli* identified in 157 samples (50.0, 95% CI 44.5–55.5). There were 135 and 187 non-duplicate ESBL-producing isolates from 2008 and 2017, respectively. In 2008, 12.6% of isolates belonged to CTX-M-1 group, all carrying *bla*_CTX-M-1_, whilst in 2017, 94.1% of isolates were CTX-M-1 group positive and of these 39.2 and 60.8% of isolates carried *bla*_CTX-M-1_ and *bla*_CTX-M-15_, respectively. In addition, the prevalence of doxycycline, gentamicin and 3rd generation cephalosporin resistance increased significantly from 2008 to 2017 while a decreased prevalence of phenotypic resistance to potentiated sulphonamides was observed.

**Conclusions:**

The real-time PCR proved a reliable and high throughput method to distinguish between *bla*_CTX-M-1_ and *bla*_CTX-M-15_. Furthermore, its use in this study demonstrated the emergence of faecal carriage of CTX-M-15 in hospitalised horses, with an increase in prevalence of ESBL-producing *E. coli* as well as increased antimicrobial resistance to frequently used antimicrobials.

## Background

Extended spectrum β-lactamase (ESBL)-producing *Escherichia coli* are typically resistant to extended spectrum cephalosporins and monobactams (aztreonam), as well as non-β-lactam agents. These bacteria are of increasing concern as ESBL encoding genes are usually harboured on plasmids, which co-harbour multiple resistance genes [1] leading to multi-drug resistance (MDR, defined as resistance to 3 or more antimicrobial classes). Clinical infections caused by MDR bacteria are particularly difficult to treat and are a leading cause of morbidity and mortality in human and veterinary medicine [2, 3]. The initially identified β-lactamase enzymes such as TEM and SHV are now becoming less prevalent in ESBL-producing *E. coli,* while CTX-M is now the most predominant mechanism in both humans and animals [4]. Almost 170 distinct ESBL CTX-M β-lactamases have been identified mostly in *Enterobacteriaceae*, including in *Escherichia*, *Klebsiella* and *Enterobacter* species [5]. The CTX-M family includes a complex group of enzymes which have been classified into five different groups; CTX-M-1, 2, 8, 9 and 25 based on their amino acid sequences [4]. These enzymes are able to efficiently hydrolyse cefotaxime and in some cases ceftazidime (CTX-M-15, CTX-M-16, CTX-M-27) [6, 7].

The worldwide emergence of the *bla*_CTX-M-15_ gene (belonging to CTX-M-1 group) during the last two decades in humans is of concern; particularly as it is often associated with the pandemic O25/ST131 *E. coli* clone [8], which belongs to the highly virulent phylogenetic group B2 and often harbours the multidrug-resistant IncFII plasmids [9]. In ST648 *E. coli* strains in human and companion animal studies, a high proportion of *bla*_CTX-M-15_ has been identified [10]. The now widespread dissemination of CTX-M-15-producing *E. coli* could have two explanations. Corresponding plasmids encoding the *bla*_CTX-M-15_ gene are being transferred via horizontal transfer to multiple lineages [11], or alternatively the strains may be spreading by clonal expansion [12].

Several studies have reported *bla*_CTX-M-1_ (also belonging to group 1) to be the most common ESBL-gene in *E. coli* in horses [13–15], however one study also identified a low prevalence of other *bla*_CTX-M_ genes [13]. CTX-M-15-producing *E. coli* has been identified in clinical isolates from horses in Germany [10, 16] and has been infrequently identified in *E. coli* from hospitalised horses in Holland [13].

Although conventional PCR assay can be used to determine which group a CTX-M enzyme belongs to, Sanger sequencing has, to date, been required to determine the CTX-M genotype [17], which has proved costly and time consuming, hence previous studies have often only classified genes to the group level. Whole genome or next generation sequencing, whilst also identifying genes and genetic contexts is still too expensive for routine surveillance. High resolution melt (HRM) analysis is an end-point real-time PCR detection method that differentiates amplicons based on their melt profile. Here we describe this method using novel primers to distinguish between different *bla*_CTX-M_ genes within group 1 based on their different melt point. While there are studies reporting the change in antimicrobial resistance (AMR) patterns in equine clinical isolates over time, there are no studies investigating carriage of AMR genes in hospitalised horse populations over time. The aims of the present study were to investigate the trend in AMR patterns over time, to determine the prevalence of *bla*_CTX-M-1_ and *bla*_CTX-M-15_ genotypes within CTX-M-1 group and to compare changes in phenotype and genotype of ESBL-producing *E. coli* over time within a single equine hospital in the UK.

## Results

In total 771 faecal samples were collected. In 2008 cohort, 457 faecal samples from 103 horses were collected with ESBL-producing *E. coli* identified in 131 samples (28.7, 95% CI 24.6–33.1) from 49 horses (47.6, 95% CI 37.7–57.6). In 2017 cohort, 314 faecal samples were collected from 72 horses with ESBL-producing *E. coli* identified in 157 samples (50.0, 95% CI 44.5–55.5) of samples from 47 horses (65.3, 95% CI 53.8–75.3). In the 2008 and 2017 cohorts, antimicrobials had been administered in the previous 7 days prior to sampling in 51.1% (67/131) and 67.5% (106/157) of samples respectively, where an ESBL-producer was identified. Antimicrobials had been administered in the previous 7 days in 32.5% (106/326) and 47.1% (74/157) of samples in the 2008 and 2017 cohorts respectively where no ESBL-producer was identified. Overall use of highest priority critically important antimicrobials (HPCIAs) was low and included 5.8% (6/103) horses (3 enrofloxacin and 3 ceftiofur) in the 2008 cohort and 9.7% (7/72) horses (2 enrofloxacin and 5 ceftiofur) in the 2017 cohort. Surgery was performed during hospitalisation in 53.4% (55/103) and 45.8% (33/72) of horses in the 2008 and 2017 cohorts respectively. The breakdown of type of cases in the two cohorts is shown in Table 1.

**Table 1:** Clinical case type in the two cohorts. GI non-surgical cases included colic cases which were treated medically. Medical cases included general medical cases such as ophthalmology, respiratory and non-GI cases. Musculoskeletal cases included mainly lameness investigation and orthopaedic surgical cases. Soft tissue cases included mass removals, dental/sinusitis investigations and surgical airway cases. GI = Gastrointestinal

Case type	2008 (*n* = 103)	2017 (*n* = 72)
GI non-surgical	5	12
GI surgical	3	8
Medical	35	9
Musculoskeletal	29	27
Soft tissue	31	16

**Fig. 1:**
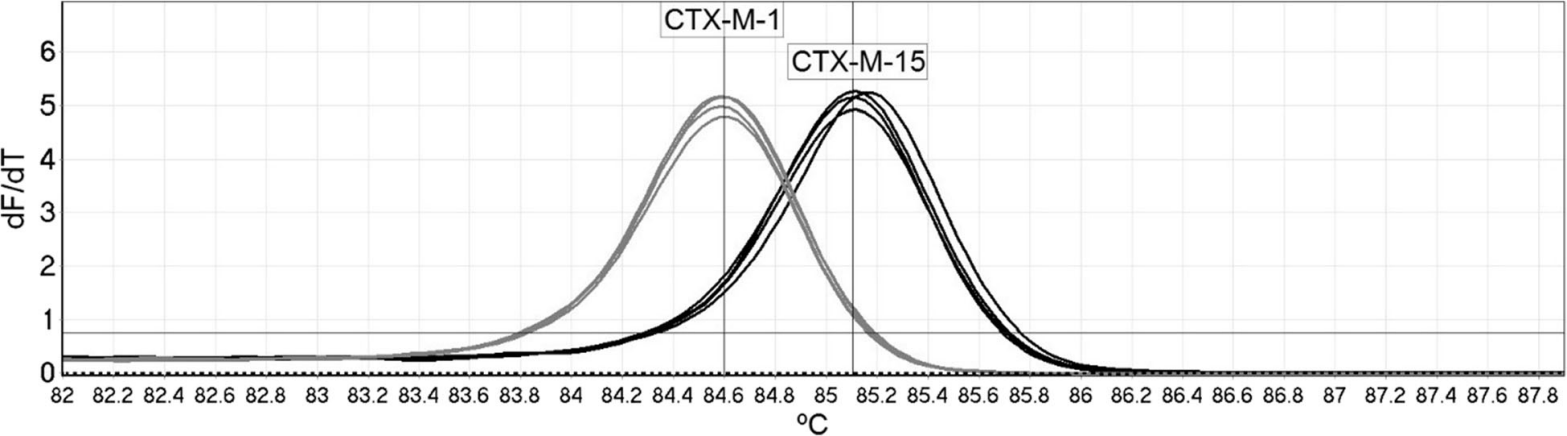
High resolution melt analysis results for four *bla*_CTX-M-1_ carrying isolates, four *bla*_CTX-M-15_ carrying isolates and a no template control. The calling threshold and calling bins for automatic genotyping are indicated by the horizontal and vertical lines, respectively

There were 135 non-duplicate ESBL isolates from 2008 cohort and 187 non-duplicate ESBL isolates from 2017 cohort. The HRM analysis demonstrated high specificity during the pilot evaluation, with no non-specific amplification generated when testing a panel of 17 non-target ESBL producers. The assay was 100% accurate at differentiating *bla*_CTX-M-1_ and *bla*_CTX-M-15_ compared with sequencing, across 44 isolates, indicating 100% agreement (Fig. 1).

Conventional PCR analysis demonstrated a reduction in carriage of *bla*_TEM_ and *bla*_SHV_ genes of approximately 50% between the two cohorts and an increase in carriage of *bla*_CTX-M_ by 37.8%. However, the *bla*_TEM_ and *bla*_SHV_ genes were not further confirmed in either cohort to determine if they were ESBL variants. In the 2008 cohort isolates, only 12.6% (*n* = 17) were CTX-M-1 group positive, all of which identified as *bla*_CTX-M-1_ using HRM analysis. In 2017 cohort isolates, 94.1% (*n* = 176) were CTX-M-1 group positive and of these 39.2% (*n* = 69) identified as *bla*_CTX-M-1_ and 60.8% (*n* = 107) as *bla*_CTX-M-15_ using HRM analysis. All 107 isolates identified as CTX-M-15 producers were negative for 025 and ST131 on PCR assay. There was a significant increase in carriage of *qnr* A (11.9%) and a small non-significant decrease in carriage of *qnr* B (− 1.26%) and *qnr* S (− 1.3%). The change in genotype between the two cohorts is shown in Table 2.

**Table 2:** The prevalence and change of genotype in ESBL-producing *E. coli* isolated from hospitalised horses in two different cohorts from the same equine hospital sampled 10 years apart

Genotype (322)	2008 cohort (*n* = 135)	Prevalence (95% CI)	2017 cohort (*n* = 187)	Prevalence (95% CI)	Change (%)	*P* -value
*bla*_SHV_ (105)	86	63.7 (55.3–71.3)	19	10.2 (6.6–15.3)	−53.5	< 0.001
*bla*_TEM_ (183)	116	85.9 (79.1–90.8)	67	35.8 (29.3–42.9)	−50.1	< 0.001
*bla*_CTX-M_ (253)	76	56.3 (47.9–64.4)	177	94.6 (90.4–97.1)	+ 37.8	< 0.001
*bla*_CTX-M_ Gr1 (193)	17	12.6 (8.0–19.2)	176	94.1 (89.8–96.7)	+ 81.5	< 0.001
CTX-M-1 (86)	17	100 (81.6–100)	69	39.2 (32.3–46.6)	−60.8	< 0.001 †
CTX-M-15 (107)	0	0 (0–18.4)	107	60.8 (53.4–67.7)	+ 60.8	
*bla*_CTX-M_ Gr2 (10)	9	6.7 (3.6–12.2)	1	0.5 (0–3.0)	−6.1	0.002 †
*bla*_CTX-M_ Gr9 (50)	50	37.0 (29.4–45.4)	0	0.0 (0.0–2.0)	−37.0	< 0.001 †
*qnr* A (27)	2	1.5 (0.4–5.2)	25	13.4 (9.2–19.0)	+ 11.9	< 0.001 †
*qnr* B (48)	30	22.2 (16.0–30.0)	18	9.6 (6.2–14.7)	−12.6	0.003
*qnr* S (19)	9	6.7 (3.6–12.2)	10	5.4 (2.9–9.6)	− 1.3	0.8

*P*-value provided for Chi-squared test apart from where † indicates use of Fisher’s Exact Test

Furthermore, there was an increased variability in phenotypic resistance in the ESBL-producing *E. coli* between the two cohorts. Some of these changes were not significant (amoxicillin, enrofloxacin and MDR), while there was a significant increase in resistance to doxycycline, gentamicin and 3rd generation cephalosporins and an increase in susceptibility to trimethoprim-sulfamethoxazole. The antimicrobial resistance in non-duplicate ESBL-producing *E. coli* is shown in Table 3.

**Table 3:** Prevalence of, and change in phenotypic AMR in ESBL-producing *E. coli* isolated from hospitalised horses in two different cohorts from the same equine hospital sampled 10 years apart (2008 and 2017) TMPS- Trimethoprim sulfamethoxazole, MDR - multidrug resistance (defined as resistance to 3 or more antimicrobial classes)

Antimicrobial agent/phenotype	2008 cohort (*n* = 135)	Prevalence (95% CI)	2017 cohort (n = 187)	Prevalence (95% CI)	% change	Total (%)	*P* value
Amoxicillin (%)	135	100 (97.2–100)	187	100 (98.0–100)	0	322 (100)	1.0 †
Cefpodoxime (%)	130	96.3 (91.6–98.4)	187	100 (98.0–100)	+ 3.7	317 (98.4)	0.01†
Ceftiofur (%)	123	91.1 (85.1–94.8)	182	97.3 (93.9–98.9)	+ 6.2	305 (94.7)	0.03
Gentamicin (%)	118	87.4 (80.8–92.0)	181	96.8 (93.2–98.5)	+ 9.4	299 (92.9)	0.0026
TMPS (%)	133	98.5 (94.8–99.6)	159	85.0 (79.2–89.4)	−13.5	292 (90.7)	< 0.001
Doxycycline (%)	53	39.3 (31.4–47.7)	172	92.0 (87.2–95.1)	+ 52.7	225 (69.9)	< 0.001
Enrofloxacin (%)	36	26.7 (19.9–34.7)	62	33.2 (26.8–40.2)	+ 6.5	98 (30.4)	0.3
MDR (%)	120	88.9 (82.5–93.2)	176	94.1 (89.8–96.7)	+ 5.2	296 (91.9)	0.1

*P*-value provided for Chi-squared test apart from where † indicates use of Fisher’s Exact Test

## Discussion

According to our current knowledge this study is the first to identify *bla*_CTX-M-15_ in ESBL-producing *E. coli* in horses at an equine hospital in the UK, as well as reporting a significant increase in prevalence of CTX-M-1 and a reduction in CTX-M-9 β-lactamase producing *E. coli* isolates in the same hospital over a decade. The study also demonstrated a significant increase in prevalence of overall carriage of ESBL-producing *E. coli* and their resistance to commonly used antimicrobials in horses, in particular, an increase in resistance to doxycycline, gentamicin and 3rd generation cephalosporins.

This is also the first study using HRM analysis to distinguish between *bla*_CTX-M-1_ and *bla*_CTX-M-15._ The assay uses a single set of primers to amplify a 213 bp region common to group 1 *bla*_CTX-M-_ genes, which contains six variant bases *bla*_CTX-M-1_ and *bla*_CTX-M-15_ that are highly conserved. The presence of these six variants results in a ~ 0.5 °C difference in the melt temperature, allowing discrimination of these genes. The ability to differentiate these genes without the requirement of any post PCR processing or sequencing provides faster results and higher throughput, will be of great benefit in studies involving large sample numbers.

There was 100% agreement between the HRM analysis and the sequencing results in the 44 pilot samples, indicating complete accuracy. HRM analysis has been used previously for applications including bacterial speciation, and the detection of resistance genes [18], and has potential both as a diagnostic test and epidemiological surveillance tool. In addition to genotyping assays, HRM analysis has also been utilised to enable highly multiplexed assays, without the use of costly hydrolysis probes [18]. HRM analysis typically has a lower analytical sensitivity than probe-based PCRs, but this is not a drawback when testing high copy number samples, such as bacterial colonies. Whilst sequencing provides a greater level of resolution, HRM analysis results were sufficient to determine the gene carried by the CTX-M producers, allowing greater number of samples to be tested during the study than if relying solely on sequencing.

Previous studies have identified *bla*_CTX-M-15_ in five clinical samples from horses in Germany [10, 16], as well as low frequency carriage (2/123 isolates, 1.6%) in hospitalised horses in Holland [13]. Our study is the first study to report CTX-M-15 β-lactamase as the predominant enzyme in ESBL-producing *E. coli* carried by horses. The emergence of CTX-M-15 β-lactamase during this 10 year period in this equine hospital is in line with the global trend of the spread of this enzyme in humans [19] and more recently in veterinary studies [16, 20, 21]. CTX-M-15 producing ST131 has also been identified in a dog in Portugal [22], and in three canine clinical isolates in the UK [23] but has yet to be identified in horses, which is supported by the results from the current study. While ST131 is rare in veterinary studies [20, 22], ST648 (phylotype D) [10] and ST410 (phylotype A) [20] have more frequently been reported from clinical and environmental hospital samples. ST410 has been recently described as a new international high-risk clone [24], whilst ESBL-producing ST648 strains have also been observed globally in human patients, as well as in chicken, pigs and wild birds in Europe [25, 26]. There was also a marked difference in the carriage of CTX-M-9 group β-lactamase producing *E. coli* isolates between the two cohorts; it was the most common group present in the first study while in the later cohort there were no CTX-M-9 ESBL-producing *E. coli* positive isolates identified, which is perhaps due to the global spread if group 1 isolates (particularly *bla*_CTX-M-15_), whilst group 9 isolates (such as *bla*_CTX-M-9_ and *bla*_CTX-M-14_), commonly found in food-producing animals [4], are becoming more scarce in the equine population [13].

The increase in prevalence of faecal-carriage of ESBL-producing *E. coli* in horses from one hospital over a decade is perhaps not a surprising finding and hospital studies involving human patients are reporting similar results [4]. The largest increase in antimicrobial resistance between the two cohorts was to doxycycline and this is a surprising finding as tetracycline use in this equine hospital is low (data not shown). Oral antimicrobials in horses are limited to potentiated sulphonamides (authorised) and enrofloxacin (no market authorisation), but more recently oral doxycycline (no market authorisation) has become a more popular treatment as it is cost-effective and can be easily administered by the owner at home. This increased popularity of doxycycline in the equine community has perhaps led to a reduction in use of potentiated sulphonamides, which may be one reason for the change in resistance patterns in these two antimicrobials in our study. There are more than 12 tetracycline resistance genes described in *E. coli* and they include three main mechanisms; drug efflux pumps, ribosomal protection proteins, and drug inactivation. Some of these genes may be plasmid mediated, in particularly *tetA* and *tetB* genes which code for efflux pumps, and the increase in tetracycline resistance may be due to co-existence on the same plasmid as the ESBL genes. Further work is required to investigate such associations, including conjugation experiments, plasmid typing and sequencing. The hospital use of HPCIAs [27], including third and fourth generation cephalosporins was low in both cohorts and there was no clear reason for the increase in prevalence of ESBL-producing *E. coli* between the two cohorts. The use of third and fourth generation cephalosporins by referring veterinarians is unknown. However, a study in 2013 identified that third and fourth generation cephalosporins only accounted for 3% of prescriptions and that equine veterinarians in first opinion practice most commonly prescribe potentiated sulphonamides [28].

Limitations of this study include the low numbers of isolates which underwent Sanger sequencing to validate the results from the HRM analysis, but in these 44 isolates there was complete agreement. Another limitation is that the study included only two sampling periods and used slightly different sampling protocols; in 2008 cohort horses were sampled every other day while in 2017 cohort horses were sampled daily, however this is unlikely to affect the results as antimicrobial susceptibility testing was repeated on the stored isolates in 2018.

## Conclusions

The present study demonstrates that HRM analysis is a reliable and low-cost method and can be used to distinguish between CTX-M-1 and CTX-M-15 β-lactamase producers in group 1 positive isolates from a conventional PCR assay without the need for sequencing of PCR amplicons. Our study also demonstrated an emergence of carriage of CTX-M-15 producing *E. coli* isolates in hospitalised horses in the UK. This may have public health implications as when these horses leave the hospital, they may contribute to the dissemination of ESBL-producing *E. coli* in the environment. It is generally accepted that carriage is a pre-requisite for infection and future studies are needed to investigate the link between faecal CTX-M-15 carriage and ESBL-producing *E. coli* obtained from clinical isolates in horses in the UK.

## Methods

Faecal samples were collected from two different cohorts of hospitalised horses at a single, large referral equine hospital in North West UK which admits approximately 1500 cases annually. Animals eligible for the study were all horses hospitalised for at least one night in the hospital. Day cases were excluded as were animals receiving chemotherapy or radiotherapy and those in isolation. Samples (~ 30 g) were taken from the top of a pile of freshly passed faeces from each horse by hospital staff, placed in sterile plastic containers and transported to the laboratory on the same site as the equine hospital. In 2008 cohort (2008–2009) faecal samples were collected every other day during hospitalisation [29], while in 2017 cohort (2016–2017), recruited from the same hospital, daily faecal samples were obtained from each horse until the horse was discharged from hospital.

### Isolation of resistant bacteria from faecal samples

*E. coli* isolation was performed according to a previously described method [29]. Briefly, 2 g of faeces were placed in a stomacher bag and 10 ml of brain heart infusion broth was added. To screen for cephalosporin-resistant *E. coli* (presumptive ESBL-producers), 0.5 ml of the faecal homogenate was also added to 4.5 ml buffered peptone water for aerobic incubation at 37 °C for 18–24 h. Following overnight enrichment, 5 μl of the faecal homogenate was streaked onto a selective media [Eosin Methylene Blue Agar (EMBA) or Harlequin agar)] containing 1 μg/ml cefotaxime using a 5 μl disposable sterile loop and incubated aerobically for 18–24 h at 37^o^ C. On the selective media, if present, two colonies resembling *E. coli* were selected from the plate and transferred onto nutrient agar and incubated overnight at 37 °C.

### Antimicrobial susceptibility and ESBL phenotypic testing

Colonies from nutrient agar plates were suspended in sterile water to make a suspension equivalent to 0.5 McFarland Turbidity Standard (0.5 MTS). Each isolate suspension was then inoculated onto a Mueller Hinton agar plates for antimicrobial susceptibility testing following the Clinical & Laboratory Standards Institute (CLSI, 2016). Double disc diffusion tests using ceftazidime (30 μg), cefotaxime (30 μg) and cefpodoxime (30 μg) ± clavulanic acid was used to confirm phenotypic ESBL-producing *E. coli.* Antimicrobial susceptibility testing was performed using discs of 10 μg amoxicillin (amox), 10 μg cefpodoxime (cpd), 30 μg ceftiofur (eft), 10 μg gentamicin (gent), 5 μg enrofloxacin (enf), 30 μg doxycycline (dxt), 1.25 μg trimethoprim + 23.75 μg sulfamethoxazole (TMPS). All microbiological media were from LabM, UK; antimicrobial agents were from Mast Group, UK (amox, cpd, gent, enf, dxt and tmps) or Oxoid, Basingstoke, UK (eft). After 16–18 h incubation at 37 °C, the bacterial growth inhibition zone diameter (mm) for each disc was measured. Isolates were categorised as susceptible if the diameter of the zone of inhibition was greater than the breakpoint for that drug [30], resulting in a binary outcome of susceptible or resistant. Control strain *E.coli* ATCC 52922 was used for susceptibility testing. MDR for *E. coli* was defined according to criteria which excludes intrinsic resistance; aminopenicillins and 3rd generation cephalosporins were considered separate classes for MDR calculations [31]. Long-term storage of isolates was undertaken at -80 °C using Microbank™ cryovials (Pro-Lab Diagnostics U.K, Cheshire UK) and isolates were recovered by removing a single bead from the cryovial using sterile forceps and inoculation of the bead onto nutrient agar for aerobic incubation at 37 °C for 16–18 h. There was 100% recovery of frozen isolates. All testing was performed by the same operators in 2017/2018 and the methods and interpretation criteria were the same for the two cohorts. Samples from 2008 were initially archived and retrieved in 2018.

### Genotypic analysis

Cell lysates were prepared by adding two to three colonies of pure 24-h cultures to 0.5 mL sterile water and heating at 100 °C for 20 min. All isolates phenotypically consistent with *E. coli* were confirmed using *uidA* gene primers in a standard PCR assay [29, 32]. All isolates confirmed as EBSL-producers using double disc diffusion test were tested by conventional PCR for *bla*_CTX-M_ genes using universal *bla*_CTX-M_ primers as previously described [33]. To determine the CTX-M group, all CTX-M positive isolates were tested using primers specific to *bla*_CTX-M_ groups 1, 2 [34] and 9 [33]. All isolates demonstrating an ESBL-phenotype were also tested for *bla*_TEM_, *bla*_SHV_ and *bla*_OXA_ encoding beta-lactamase genes [35] and plasmid-mediated *qnrA, B and S* genes conferring quinolone resistance using further multiplex PCR assays [36].

### HRM analysis

All isolates identified as carrying CTX-M-1 group ESBL genes were categorised as *bla*_CTX-M-1_ or *bla*_CTX-M-15_ producers using a novel HRM analysis based real time PCR assay. Primers were designed from CTX-M-1 group sequences aligned in MEGA, using ClustalX. A 213 bp region was selected with the maximum variation between *bla*_CTX-M-1_ or *bla*_CTX-M-15_ gene sequences, to allow for the greatest Tm shift during melt analysis. Primers were designed using Primer3 (http://primer3.ut.ee/), and amplicon Tm was estimated using the nearest neighbour method in OligoCalc (http://biotools.nubic.northwestern.edu/OligoCalc.html). *E. coli* 13353 was used as a control strain in the assay validation (CTX-M-15). The primer details are shown in Table 4.

**Table 4:** Forward and reverse sequence, amplicon size and melt temperature for the high-resolution melt curve analysis distinguishing between *bla*_CTX-M-1_ or *bla*_CTX-M-15_ within CTX-M group 1

Primer	Sequence 5 > 3	Amplicon size	Melt Tm
CTXMG1 F	TGTTGTTAGGAAGTGTGCCG	213 bp	84.75 C (CTX-M-1)
CTXMG1 R	CGCTTTCACTTTTCTTCAGCAC		85.25 (CTX-M-15)

Reactions were carried out in the Rotor-Gene Q 6000 (Qiagen, Germany) using Type-It HRM mix (Qiagen, Germany), and 400 nM of both forward and reverse primers. Cycling conditions were 95 °C for 5 min, followed by 30 cycles of 95 °C for 10 s, 58 °C for 30 s and 72 °C for 10 s. Reactions were monitored in the FAM channel, and end point high resolution melt analysis was carried out using the HRM channel at 0.1 °C increments from 72 °C to 95 °C. The calling bins for *bla*_CTX-M-1_ and *bla*_CTX-M-15_ gene were set at 84.75 °C (+/− 0.1 °C) and 85.25 °C (+/− 0.1 °C), respectively, by the predicted melt temperatures. The specificity of the primers was confirmed by testing against a bank of 17 non-target β-lactamase producers, including CTX-M-9 (*n* = 5), CTX-M-2 (*n* = 1), CTX-M-8 (n = 1) group, TEM (n = 5) and SHV (n = 5) positive isolates. The accuracy of the melt analysis method was validated by Sanger sequencing (Source Bioscience, UK), where the *bla*_CTX-M_ gene was confirmed in 44 CTX-M-1 group carrying isolates (9 *bla*_CTX-M-1_, 35 *bla*_CTX-M-15_) and then testing these isolates using HRM analysis.

The assay was then used to test all CTX-M-1 group producers identified in the study. Isolates carrying *bla*_CTX-M-15_ were tested further by conventional PCR assay to determine whether they belonged to serogroup O25 [37] and for markers for sequence type (ST)131 [38], representing the pandemic *E. coli* clone associated with CTX-M-15 in human clinical infections [8].

### Data analysis

The 95% confidence intervals around proportions were calculated using Wilson’ Score interval [39]. Differences in proportions between the two cohorts were compared using a Chi-squared test. Where a comparison had less than five in any category a Fishers exact test was used. All analysis was performed using EpiTools [39]. A *P* value of < 0.05 was regarded as significant.

## Data Availability

The datasets used and analysed during the current study are available from the corresponding authors on reasonable request.

## Abbreviations

AMR: Antimicrobials resistance
CLSI: Clinical & Laboratory Standards Institute
EMBA: Eosin methylene blue agar
ESBL: Extended spectrum β-lactamase
HRM: High resolution melt
MDR: Multi drug resistance
MTS: McFarland turbidity standard
PCR: Polymerase chain reaction
TMPS: Trimethoprim sulfamethoxazole
